# Introgression of Maize Diversity for Drought Tolerance: Subtropical Maize Landraces as Source of New Positive Variants

**DOI:** 10.3389/fpls.2021.691211

**Published:** 2021-09-23

**Authors:** Pedro Augusto Medeiros Barbosa, Roberto Fritsche-Neto, Marcela Carvalho Andrade, César Daniel Petroli, Juan Burgueño, Giovanni Galli, Martha C. Willcox, Kai Sonder, Víctor A. Vidal-Martínez, Ernesto Sifuentes-Ibarra, Terence Luke Molnar

**Affiliations:** ^1^Allogamous Plant Breeding Laboratory, Department of Genetics, Luiz de Queiroz College of Agriculture, University of São Paulo, Piracicaba, Brazil; ^2^International Maize and Wheat Improvement Center (CIMMYT), Texcoco, Mexico; ^3^Instituto Nacional de Investigaciones Forestales, Agrícolas y Pecuarias (INIFAP), Mexico City, Mexico

**Keywords:** plant breeding, GWAS, spatial analysis, abiotic stress, early testcross

## Abstract

Current climate change models predict an increased frequency and intensity of drought for much of the developing world within the next 30 years. These events will negatively affect maize yields, potentially leading to economic and social instability in many smallholder farming communities. Knowledge about the genetic resources available for traits related to drought tolerance has great importance in developing breeding program strategies. The aim of this research was to study a maize landrace introgression panel to identify chromosomal regions associated with a drought tolerance index. For that, we performed Genome-Wide Association Study (GWAS) on 1326 landrace progenies developed by the CIMMYT Genetic Resources Program, originating from 20 landraces populations collected in arid regions. Phenotypic data were obtained from early testcross trials conducted in three sites and two contrasting irrigation environments, full irrigation (well-watered) and reduced irrigation (drought). The populations were genotyped using the DArTSeq^®^ platform, and a final set of 5,695 SNPs markers was used. The genotypic values were estimated using spatial adjustment in a two-stage analysis. First, we performed the individual analysis for each site/irrigation treatment combination. The best linear unbiased estimates (BLUEs) were used to calculate the Harmonic Mean of Relative Performance (HMRP) as a drought tolerance index for each testcross. The second stage was a joint analysis, which was performed using the HMRP to obtain the best linear unbiased predictions (BLUPs) of the index for each genotype. Then, GWAS was performed to determine the marker-index associations and the marker-Grain Yield (GY) associations for the two irrigation treatments. We detected two significant markers associated with the drought-tolerance index, four associated with GY in drought condition, and other four associated with GY in irrigated conditions each. Although each of these markers explained less than 0.1% of the phenotypic variation for the index and GY, we found two genes likely related to the plant response to drought stress. For these markers, alleles from landraces provide a slightly higher yield under drought conditions. Our results indicate that the positive diversity delivered by landraces are still present on the backcrosses and this is a potential breeding strategy for improving maize for drought tolerance and for trait introgression bringing new superior allelic diversity from landraces to breeding populations.

## Introduction

Maize is a major crop cultivated in many regions of the world and is one of the most important cereals for food production, occupying around 193 million hectares in 2018 (Faostat, 2018). That corresponds to about 15% of the total area in agricultural use. Globally, the majority of maize hectares planted are in subtropical and tropical regions including many parts of Latin America, Africa, and Asia (Faostat, 2018). Millions of smallholders and subsistence farming communities in these regions rely on maize as a major source of calories as well as income to pay for basic needs and schooling for their children. For optimal yields, maize demands a large amount of water; therefore, drought events at key junctures in the growing cycle can cause significant losses in grain yield and year-to-year yield fluctuations (Harrison et al., 2014). This yield instability creates great financial incertitude in the farming communities of developing countries where maize is important. Unfortunately, most climate change forecast models concur that an increase in the frequency and intensity of drought events is occurring and will continue to occur throughout the 21st century in many global regions (IPCC, 2007, 2014; Dai, 2013; Hoegh-Guldberg et al., 2018; Lu et al., 2019; Spinoni et al., 2020). These data suggest that if maize varieties with novel sources of drought tolerance are not soon developed, smallholder and subsistence farmers in many parts of the world will become even more vulnerable to yield fluctuations and the financial insecurity that entails. Fortunately, maize is a species with immense genetic variability and breeding for drought tolerance is an essential tool for overcoming the effects of climate change in maize production (Fisher et al., 2015). This genetic diversity has largely been collected in the world’s germplasm banks and is additionally evident in the fields of many smallholder farmers that still plant landrace populations (Sanchez et al., 2000; Prasanna, 2012; Arteaga et al., 2016). In this context, the more than 25,000 maize landrace accessions maintained in the germplasm bank of the International Maize and Wheat Improvement Center (Spanish acronym, CIMMYT) are a tremendous resource for identifying novel resistance and developing new cultivars better able to maintain grain production in water-limited conditions. The reality, however, is that maize breeders in the private and public sector have for many years not used as new sources of variation the landrace accessions held in germplasm banks. This is mostly due to such factors as linkage drag, adaptation issues, poor agronomic characteristics and low yield potential (Goodman, 1985; Goodman et al., 2014). For that reason, in 2011 CIMMYT initiated the Seeds of Discovery project (SeeD), one of the components of MasAgro initiative, which has been largely funded by the Mexican government. The objective of SeeD called later MasAgro Biodiversidad, is to mine the unexplored allelic variation in the maize germplasm bank for important abiotic and biotic pressure as well as nutritional and end-use characteristics.

Under abiotic stress conditions, tolerance is the ability of the plant and its yield component traits to maintain grain production during an extended period under that stress (Schafer, 1971; Miti et al., 2010). Between stand establishment and physiological maturity, maize has two periods in the growth cycle when the crop is most vulnerable to yield loss due to drought conditions: (i) from the late vegetative growth stage through to the end of flowering and (ii) during the grain filling period (Sayadi Maazou et al., 2016). Of these two, the flowering stress is considered the more devastating because of the greatly increased anthesis-silk interval (ASI) that can be provoked by drought conditions (Bolaños and Edmeades, 1996). ASI measures the number of days from when anthesis occurs to when receptive silks (stigmas) emerge. In non-stressed conditions, the maize male inflorescence typically begins releasing pollen one to two days before silk emergence and releases pollen for 4–7 days, existing a considerable variability in this period across genotypes (Hall et al., 1982; Struik and Makonnen, 1992; Uribelarrea et al., 2002). However, under drought stress the interval between anthesis and silk emergence can be greatly increased resulting in significantly reduced yield because a reduced amount viable pollen is available when the silks finally do emerge. In more extreme drought conditions, silks may emerge too late to receive pollen or may not emerge at all resulting in barren plants and no grain production. Because of this vulnerability, ASI is one of the most studied traits in maize, and it has been used as a secondary trait for indirect selection for grain yield and drought tolerance (Bolaños and Edmeades, 1996; Xue et al., 2013). CIMMYT has conducted long-term recurrent selection breeding in two populations for drought tolerance focused on decreasing ASI and maintaining grain yield under drought stress (Bänziger et al., 2000). These two populations, La Posta Sequía (LPS) formed in the mid-1980s, and Drought Tolerant Population (DTP) formed in the early 1990s have now gone through recurrent selection for 7 and 9 cycles, respectively. In total, fifteen official CIMMYT maize inbred lines (CML) and several open-pollinated varieties (OPV) have been derived and released from the various cycles of LPS and DTP. These CML and OPV have been used extensively in east Africa, South Asia and Latin America and are adapted to either highland, mid-altitude subtropical or lowland tropical environments. While this breeding project was successful in developing new sources of elite drought tolerant material, the LPS and DTP source material represents only a fraction of the overall diversity in the CIMMYT germplasm bank. Much more of this diversity needs to be explored, using the latest techniques to unravel the genetic control of drought tolerance in maize. And while ASI remains a trait of importance in developing drought tolerant maize, a plant’s response to drought stress is based on physiological and morphological modifications that involve a complex number of genes and molecular pathways (Rengel et al., 2012; Min et al., 2016). By better comprehension, the genetics underlying drought tolerance, and by accessing more of the genetic diversity available for this trait, breeding programs working on this trait will improve selection precision and the speed of breeding cycles.

In order to understand both the genetic diversity of a specie and the complex genetic factors controlling response to abiotic stress, molecular markers are an important tool for geneticists and breeders (Xu et al., 2017). Methodologies such as genome-wide association studies (GWAS) seek to relate molecular markers to observed phenotypes and thus discover the chromosomal regions associated with the trait of interest. In maize, this procedure has been used for many different traits, both abiotic and biotic (Xiao et al., 2017). In others important cultivated plants, GWAS has been used in studies on millet (Jaiswal et al., 2019), common beans (Fritsche-Neto et al., 2019), rice (Huang et al., 2010), soybeans (Hwang et al., 2014). In a review on the use of GWAS in maize, Xiao et al. (2017) showed that most studies use a panel of inbred lines with *per se* phenotypic data or, seldomly, testcross hybrids. For crops such as maize where hybrids are used to exploit heterosis, the testcross is an important strategy in research for the development of improved hybrids, as it represents the real genotypic constitution of the crop grown by farmers. Additionally, early generation testing (EGT) of semi-inbred lines as testcrosses is a common strategy used in maize breeding programs, being a cross between a tester inbred or F_1_ with a S_2_ or S_3_ experimental line. The aim is to take advantage of early selection for yield potential, avoiding future testing costs on unpromising families (Bernardo, 2010). EGT takes advantage of the fact that yield potential can be identified early in the inbreeding process and shortens the time to line release because the inbreeding and testing process can be done in tandem instead of sequentially. Early testcross populations to date have not been commonly used in GWAS studies. Therefore, this study aims to identify genomic regions related to drought tolerance in two sequential inbreeding generations of early testcrosses obtained between landrace-derived semi-inbred lines and elite testers. The main reason to use landraces is to bring novel positive alleles to a breeding population, we intend to do that combining some breeding, genetics, and analytics techniques, as back-cross, early-testcross, GWAS, selection index and mixed models.

## Materials and Methods

### Plant Material

Before the evaluations discussed in this study, 326 landraces of subtropical adaptation were evaluated in *per se* drought trials over 2 years (data not shown, see https://seedsofdiscovery.org/catalogue/maize-drought-tolerant-landraces/ for more details). Subtropical adapted maize landraces are from areas between 800 and 1800 m above sea level (Edmeades et al., 2017). The 326 were selected from the CIMMYT International Germplasm Bank and the Mexican National Maize Collection collected and managed by Instituto Nacional de Investigaciones Forestales, Agricolas y Pecuarias (INIFAP) using an aridity index described in Ruiz Corral et al. (2013). They originated from dryland production areas, with 290 (89%) coming from Mexico and the rest from Argentina and Chile. At least 24 different maize races were represented in the selected set of accessions, however, 17% of them were not racially classified. Although latitude also plays an important role in adaptation, we did not consider latitude in the selection of the landraces as drought evaluations in Mexico are conducted during the winter dry season and much of the photoperiod sensitivity that could potentially be provoked by a change in latitude is negated. Based on the results of the *per se* evaluations, the 20 highest performing accessions were used to develop semi-inbred lines by the SeeD maize breeding project in the Genetic Resources Program of CIMMYT.

**FIGURE 1:**
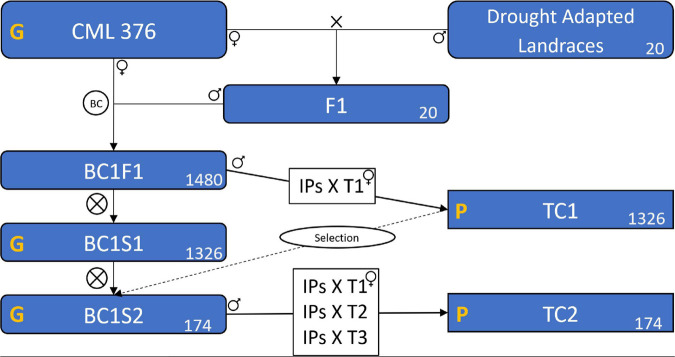
Scheme of the plant material formation showing the populations, the crosses, and the testcrosses. CML 376 is the maize line used as recurrent parental. The X means a cross. BC represents the backcross between the F1s and the CML 376. BC1F1 is the population resulted from the backcross, BC1S1 is the result of BC1F1 self-pollination, likewise, BC1S2 is the population resulted from BC1S1 self-pollination. The ⊗ means a self-pollination. TC1 and TC2 are the two testcross trials carried as field experiments. IPs is Individual Plants of the BC1F1 and BC1S2 that were crossed with the tester(s). T1, T2, and T3 are the three tester genotypes. The yellow G means that the population was genotyped, and P means that the field trials were phenotyped. The number in the lower left corner of the boxes is the number of genotypes, or populations in the case of Landraces and F1. The “Selection” on a dashed line means that we used the results of TC1 to select the superior BC1S1 genotypes, this process selected the 174 superior genotypes among the 1326 existing in BC1S1, the selected genotypes formed the BC1S2.

In deriving inbred lines from maize landraces, crossing to an elite line is a common practice to avoid severe inbreeding depression and to add favorable alleles for important agronomic traits (Tarter et al., 2004; Meseka et al., 2015; Navarro et al., 2017). In this case, the 20 selected landraces were crossed with the elite CIMMYT line CML376 to generate 20 segregating F1 populations (Figure 1). The F1 populations were then crossed again to CML376 (backcross) and ears were harvested and shelled individually creating 1480 BC1F1 lines with an average of 74 lines per landrace population and each line averaging 25% landrace genome. In the following cycle, individual plants were selfed to produce BC1S1 lines and at the same time the plant was crossed to the F1 tester CML264/CML311 (hybrid produced by crossing CML264 and CML311) to produce testcross seed for yield trial evaluations (TC1) in the winter of 2015–2016. This tester was selected because it is a commonly used subtropical tester in Mexico and has the advantage of producing large quantities of testcross seed. Based on their TC1 yield performance under drought and well-watered conditions, 68 entries were selected for further testing (Table 1). The BC1S1 lines had been selfed in a nursery while the yield trials were conducted but only 64 of them produced harvestable BC1S2 ears. Two to three ears per BC1S1 line were harvested for a total of 174 BC1S2 ears. In the summer, these 174 BC1S2 lines were crossed to 3 testers for TC2 trials in the winter of 2016–2017. The testers used were the TC1 tester, CML264/CML311, plus CML373 and CL501801.

**TABLE 1:** 20 Landraces from CIMMYT germplasm bank used as a source for the breeding populations studied in this research, number of progenies of each landrace evaluated in generation 1 (backcross1, selfing 1 - BC1S1) and generation 2 (backcross 1, selfing 2 - BC1S2).

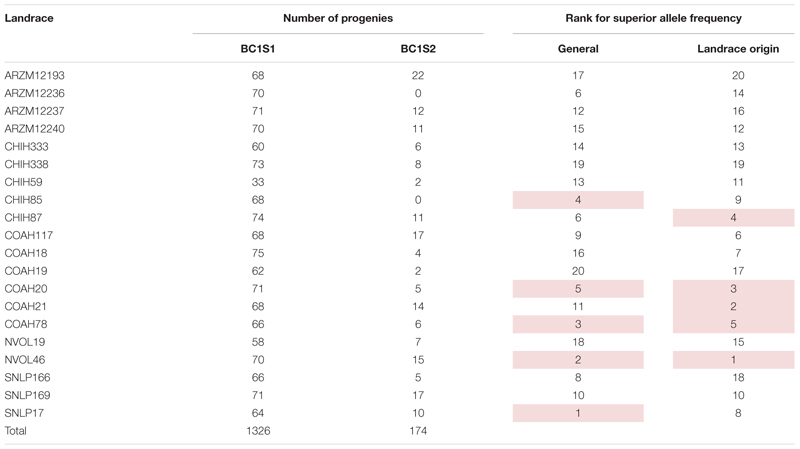

*The rank of allele frequency of superior alleles considering the 10 significant SNPs (General) and the 4 SNPs with superior allele originated from landraces (Landrace origin), 1 is the first position, i.e., with the highest frequency and 20 the last, with the lowest.*

*Highlighted the top five landraces for each rank of superior alleles frequency.*

### Testcross Phenotyping and Experimental Designs

From the 1480 BC1F1 individual plants selfed and crossed to the tester CML264/CML311, 1326 produced sufficient seed quantities of both selfed seed and testcross seed. Six check hybrids were added, so the TC1 experiment had a total of 1332 entries. The TC1 was carried out at three locations: the INIFAP experiment station at Santiago del Ixcuintla (SI) – Nayarit State, the CIMMYT experiment station at Tlaltizapán (TL) – Morelos State and the INIFAP station at Los Mochis (LM) – Sinaloa State. In each location there were two treatments, drought (DR) and well-watered (WW). A sparse phenotyping arrangement of genotypes in location was used due to restrictions on field space and seed amount, but the landraces ancestry was balanced in each location. That means, we did not test the entire set of 1332 genotypes in all locations, using so an unbalanced distribution of genotypes across location. The distribution of the testcross entries was: 266 tested only in TL (∼13 progenies of each 20 landraces), 266 tested only in LM (∼13 of each 20 landraces), 265 tested in TL and LM (∼13 of each 20 landraces), 529 tested in the 3 locations (∼26 of each 20 landraces). Thus, the TC1 experiment consisted of 6 combinations of location and treatment (3 locations and 2 irrigation treatments). The experiment was designed in a way that each location contains three plots of the tested germplasm in that specific location, one plot in irrigated condition and two plots in drought condition. The irrigated (WW) treatments were planted in a modified augmented design with systematically distributed spatial check plots arranged in a chess knight move, the field implementation of this design were based mainly on the studies of Cullis et al. (1998); Muller et al. (2010), and Clarke and Stefanova (2011). The percentage of checks ranged from 11.7% in LM, 13.7% in TL, and 20.4% in SI. The DR treatments were conducted in a randomized complete block design with two replicates, except in Santiago del Ixcuintla, where even the DR experiment was conducted as the WW experiments. The checks included in the trial were classified into three different categories: (i) the genetic check consisting of the recurrent parent crossed to the tester, CML264/CML311//CML376; (ii) the comparative checks consisting of the commercially sold hybrid CML264/CML311//CL106951 and the CIMMYT standard check for drought evaluations, LPSC7-F64/CML550; (iii) and finally, the spatial checks, hybrids CEBU, P3055W, and P4082W.

The TC1 trials were planted on January 14, 20, and 30 of 2016 at TL, SI, and LM, respectively. Harvest dates for the trial, in the same order, were June 4, 10, and 27. Winter planting dates were chosen because this is the dry season for much of Mexico, especially the western half of the country. Using drip irrigation in all the sites, we were able manage the amount of water delivered to each treatment. In all locations we used a two-row plot, in TL and SI were 4.5 m long with 0.75 m between the rows, and in LM, 4 m rows spaced by 1 m. The agronomic treatments (fertilizers, insecticides, and herbicides) were applied following common local practices. The irrigation in the drought treatments was interrupted 3 weeks before the expected anthesis date at each location, and after flowering terminated one more irrigation was done to ensure the grain filling. The plots were harvested by hand, the ears threshed by machine, the weight and moisture grain of each plot were taken, and the plot grain production was adjusted to 13% of grain moisture and converted to 1000 kg per hectare (Mg.ha^–1^), considering the differences in plots size at each site.

The TC2 trials followed a similar protocol. The set of 174 BC1S2 lines selected using the TC1 data were tested in 18 location × treatment × tester combinations (3 locations, 2 irrigation treatments, and 3 testers. The 3 locations were TL, LM, and San Juan de Abajo (PV) – Nayarit State, replacing the SI TC1 location, the 2 irrigation treatments were WW and DR, and the 3 testers were the hybrid CML264/CML311 and the lines CML373 and CL501801. All TC2 trials used two-rows plot of 4 m length, with 0.75 m between rows, harvest was accomplished using a Wintersteiger Classic plot combine at TL and PV location, and a New Holland TR 88 2-plot combine at LM, both combines provide measures of the grain weight and grain moisture of each plot. The agronomic treatments and water supply were managed the same as for the TC1 trials.

### Genotypic Data

We used DArTseq technology (Sansaloni et al., 2011), developed by Diversity Array Technology company,^1^ for genotyping all the 1326 BC1S1 and 174 BC1S2 lines and the recurrent parent CML376. The genotyping was carried out in the Genetic Analysis Service for Agriculture (Spanish acronym, SAGA) facility at CIMMYT, Mexico. A bulk of 10 seeds of each BC1S1 and BC1S2 line was sampled. A genomic representation was generated by digesting nuclear DNA with a combination of two restriction enzymes, *Pst*I (CTGCAG) and *Nsp*I (CATG), and ligating individual barcodes adapters to identify the origin of each fragment after a samples pooling. Successfully amplified fragments were sequenced using the sequencer Illumina HiSeq2500 (Illumina Inc., San Diego, CA). Then, the SNP calling for those fragments was produced by the DArTsoft analytical pipeline (DArTsoft; DArT P/L, Australia^2^) where the markers were identify *de novo* by comparing the sequences of fragments present in genomic libraries of samples previously processed, this process identifies and calls SNP markers against an existing library and it is totally independently of any reference genome. After this process, a set of filtering parameters were then applied to select high-quality markers for this study. To obtain markers positions on the chromosomes, the DNA fragment sequences were BLASTed against the *Zea mays L.* reference genome (**B73 RefGen_v4**). This procedure resulted in a genomic profile of 47,047 SNPs. Of this total, the markers of uncertain mapping or non-nuclear were still discarded, resulting in a profile of 32,592 SNPs with excellent quality that were positioned on the maize reference genome.

The 32,592 SNPs were named, for this study, by their order in the genome from M1 (the first mark in chromosome number 1) to M32592 (the last marker in chromosome number 10). A final quality control procedure was applied, and markers with minor allele frequency (MAF) <5% were excluded and a call rate ≥95% used. Thus, markers with more than 5% of missing data were excluded. The imputation of missing data was performed by the Wright method carried out using the R package snpReady (Granato and Fritsche-Neto, 2018) using the function raw.data, and resulted in a final total of 5,695 SNPs (Supplementary Table 1). The genome coverage was obtained by calculating the SNP density within a 100 Mb window, that is, the number of SNPs in each 100 Mb DNA segment for all the chromosomes, for this was used the R package rMVP (Yin et al., 2020).

### Phenotypic Analysis

For analysis of the phenotypic data, a two-stage process was performed. In the first stage, adjusted yield means of genotypes were obtained by location, and then in the second stage a mixed model was fit jointly considering residual weights estimated in the first stage. The spatial corrections were applied in the first stage, using a first-order autoregressive process (AR1⊗AR1) to model the covariance structure of error. The spatial modeling approach was chosen due to some factors as the high number of genotypes tested, the augmented design with repeated check plots applied and the large size of the field trial.

The WW treatments were designed in a complete spatial model, with no replicates of the test genotypes, and the replicated checks were systematically distributed in the field, so these trials were fitted with the following model in the first stage:

y=X⁢τ+e

where *y* is the vector of phenotypic data, arranged by Row x Column (plot field position), *X* is the design matrix of fixed effects; τ is the vector genotypes effects (and tester in the case of TC2 trials), and eventual spatial factors row and columns (as explained below); and *e* vector of errors [*e*∼*N*(0,*R*σ^2^)]; ***R*** is the covariance matrix of *e*. The spatial analysis does a decomposition of the *e* into a vector ζ of spatial correlated effects, and a vector η of spatially independent residuals, in this spatial approach of modeling the residual we assume the error variance as:

R=v⁢a⁢r⁢[ζ]+v⁢a⁢r⁢[η]

The *var*[η] is assumed to be independent (σ^2^*I*_*n*_), and the *var*[ζ] is the correlated spatial error, and we assume a first-order autoregressive, (AR1⊗AR1) (Gilmour et al., 1997), so the error variance is the sum of them, and we can show it as (Dutkowski et al., 2002):

$$R=\sigma_{\zeta }^{2}\,\left[A\,R\,1\,\left(\rho_{c\,o\,l}\right)⊗A\,R\,1\,\left(\rho_{r\,o\,w}\right)\right]+\sigma_{\eta }^{2}\,I$$

where AR1(ρ_*col*_) and AR1(ρ_*row*_) are the first-order autoregressive correlation matrices for columns and rows. The components $\sigma_{\zeta }^{2}$ and $\sigma_{\eta }^{2}$ are the spatial residual variance and the non-spatial residual variance, respectively, ⊗ is the Kronecker product. The (ρ_*col*_) and (ρ_*row*_) are the autocorrelation parameter for field columns and field rows:

$$\text{AR}\,1_{\text{row}}=\left[\begin{array}{ccccc} 1 & \rho_{\text{row}} & \rho_{\text{row}}^{2} & \cdots & \rho_{\text{row}}^{r-1}\\ \rho_{\text{row}} & 1 & \rho_{\text{row}} & \cdots & \vdots \\ \rho_{\text{row}}^{2} & \rho_{\text{row}} & 1 & \cdots & \vdots \\ \vdots & \vdots & \vdots & \ddots & \vdots \\ \rho_{\text{row}}^{r-1} & \cdots & \cdots & \cdots & 1 \end{array}\right]$$

$$\text{AR}\,1_{\text{col}}=\left[\begin{array}{ccccc} 1 & \rho_{\text{col}} & \rho_{\text{col}}^{2} & \cdots & \rho_{\text{col}}^{c-1}\\ \rho_{\text{col}} & 1 & \rho_{\text{col}} & \cdots & \vdots \\ \rho_{\text{col}}^{2} & \rho_{\text{col}} & 1 & \cdots & \vdots \\ \vdots & \vdots & \vdots & \ddots & \vdots \\ \rho_{\text{col}}^{c-1} & \cdots & \cdots & \cdots & 1 \end{array}\right]$$

The Kronecker product of them results in the symmetric correlation matrix (size n by n, where n is the number of plots) that captures the correlation between each pair of plots.

$$\text{AR}\,1_{\text{row}}⊗\text{AR}\,1_{\text{col}}=\left[\begin{array}{cccc} 1 & c\,o\,r_{1,2} & \cdots & c\,o\,r_{1,n}\\ c\,o\,r_{2,1} & 1 & \cdots & c\,o\,r_{2,n}\\ \vdots & \vdots & \ddots & \vdots \\ c\,o\,r_{n,1} & c\,o\,r_{n,2} & \cdots & 1 \end{array}\right]$$

Where *cor*_*1,2*_ is the correlation between the plot 1 and plot 2, and η is the number of plots. Therefore, as said before, the final error variance structure for the first stage models is the sum of the correlated spatial error and the spatial independent error:

$$\text{R}=\sigma_{\zeta }^{2}\,\left[\begin{array}{cccc} 1 & c\,o\,r_{1,2} & \cdots & c\,o\,r_{1,n}\\ c\,o\,r_{2,1} & 1 & \cdots & c\,o\,r_{2,n}\\ \vdots & \vdots & \ddots & \vdots \\ c\,o\,r_{n,1} & c\,o\,r_{n,2} & \cdots & 1 \end{array}\right]+\sigma_{\eta }^{2}\,I_{\left(n\,x\,n\right)}$$

The DR treatments follow the same logic for the error variance structure. The difference between the analysis of WW treatments and DR treatments is in the fixed section of the model, in the drought management there were replicates, so the factor replicate was added as fixed effects. Therefore, the model is similar, but *X* also contains the block design, and the vector τ also contains the fixed effects of replicates.

This analysis provides a variogram plot of the residuals that shows in a geographic form any spatial pattern that may be a result of systematic variation due to the row and columns effects. Depending on the variogram of each trial, the row and column may be included as a fixed effect factor (Burgueño et al., 2000). The Wald test was used to verify the significance of adding this fixed effect in the model. And when significant they were retained in the model.

Once all the individual trials were fitted to its specific model, we obtained the genotypes adjusted means (BLUE) of each location and irrigation treatment irrigation treatment.

### Harmonic Mean Relative Performance Index

With the adjusted mean of each genotype in each combination of location and irrigation treatment irrigation treatment, we applied the Harmonic Mean of Relative Performance Index (HMRP) as a measure of genotype drought tolerance in each location. This index, proposed by Resende (2004), allows selection of genotypes that had good performance in both contrasting environments, in our case, in the drought and well-watered fields. The following equation is used to calculate the HMRP index:

$$\text{HMR}\,P_{i\,j}=\frac{2}{{\left(\frac{G\,Y\,w\,w_{i\,j}}{{\bar{y}}_{w\,w_{j}}}\right)}^{-1}+{\left(\frac{G\,Y\,d\,r_{i\,j}}{{\bar{y}}_{d\,r_{j}}}\right)}^{-1}}$$

where *HMRP*_*ij*_ is the harmonic mean relative performance of genotype *i* in site *j*; *GYww*_*ij*_ is the BLUE of grain yield under well-irrigation treatment of genotype *i* in the site *j*; *GYdr*_*ij*_ is the BLUE mean of grain yield in the drought condition of genotype *i* in site *j*; ${\bar{y}}_{w\,w\,j}$ and ${\bar{y}}_{d\,r\,j}$ are the overall BLUE means of the well-watered and drought condition experiments, respectively, in site *j.*

For the joint analysis (second stage), we used a linear mixed model to compute the best linear unbiased predictions (BLUPs) of the HMRP, grain yield in drought (GYDR) and well-watered (GYWW) conditions for each genotype fitting the following model:

y=X⁢β+Zg+Wi+e

where *y* is the vector of BLUEs obtained in the first stage; β is the vector of fixed effects of site, *g* is the vector of the genotypic values of the lines, *i* is the vector of the random effects of the interaction sites by genotype, *e* is the random errors vector. *X*, *Z*, and *W* are the design matrix for β, *g*, and *i*. The weighting method was based on squared standard errors from stage one BLUES. As the weight of HMRP, we used the mean of squared standard errors of the well-watered and drought experiments, as the index use both adjusted means (WW and DR BLUES) as its components. The significance of the fixed effect of location was estimated using Wald statistic, and for the random effects, genotypes, and genotypes x location interaction, by Likelihood Ratio Test (LRT). The broad-sense heritability, for the joint analysis, was estimated as:

$$h^{2}=\frac{\sigma_{g}^{2}}{\sigma_{g}^{2}+\frac{\sigma_{g\,e}^{2}}{l}+\frac{\sigma_{e}^{2}}{l\,r}}$$

where $\sigma_{g}^{2}$ is the genotypic variance, $\sigma_{g\,e}^{2}$ is the variance due to genotype by site interaction, $\sigma_{e}^{2}$ is the residual variance, *l* is the number of sites, 3, in this study and *r* is the number of replicates. All the phenotypic analyses were performed using the ASReml-R v.3 package (Butler, 2009) in R, version 3.5.3 (R Core Team, 2019). In the Supplementary Material you can find R codes and some comments on how these analyses were carried out.

### Genome-Wide Association Studies

Genome-wide association analysis based on Mixed Linear Model (MLM) was performed using the Fixed and Random Model Circulating Probability Unification in the R package FARMCPU (Liu et al., 2016) for three traits: grain yield in drought and well-watered conditions and the drought tolerance index (HMRP). Principal Component Analysis was done using genomic information to assess population structure. The genome-wide association model (MLM equation) used was:

g=X⁢β+Zu+e

where *g* is the vector of adjusted phenotypic observation (BLUPs of HMRP obtained in the joint analysis presented above, or GYDR and GYWW); β is the vector of the fixed effects of intercept, single markers, and the three first principal components used for population structure control; *u* is the vector of random additive effects; *e* is the vector of random residual; the *X* is the design matrix of fixed effects; *Z* is the genotype incidence matrix driven by VanRaden’s genomic relationship matrix (GRM) (VanRaden, 2008), so *u*∼N(0,**G**), where G is the VanRaden’s GRM. A Multiple-test threshold adjustment was performed by a permutation method to determine the SNP significance level. This procedure was carried out in FARMCPU using the FarmCPU.P. Threshold function. The same GWAS process was done with the BC1S1 and BC1S2 populations, using the phenotypic data from TC1 and TC2, respectively.

The linkage disequilibrium for the BC1S1 data was measured by the *r*^2^ parameter (square of the correlation coefficient between two loci), using Synbreed R package (Wimmer et al., 2012). The LD decay pattern for each chromosome was evaluated by non-linear regression fitting expectation of *r*^2^ of each pair of markers with the distance between them to generate a curve of LD decay (Hill and Weir, 1988; Remington et al., 2001).

### Gene Annotation

Through the MaizeGDB (Portwood et al., 2019) and National Center for Biotechnology Information (NCBI^3^) databases, a search into maize reference genome (B73 RefGen_v4) was carried out to check the surroundings of GWAS-significant markers. This search was done inside a window of 200k base pairs (bp) centered on the markers, i.e., in a chromosome segment of 100k bp going up and downstream of the markers, we used this window size based on the LD decay obtained to our population and on the maize LD decay reported in the literature. Gene ontology is not thoroughly consistent across platforms, and MaizeGDB notation was chosen as primary. The functional annotation of the genes found inside those windows was analyzed and related to a drought tolerance process.

### Origin of Alleles for Significant Markers and Superior Allele

The purpose of using landraces in a breeding program is to bring novel positive alleles to a breeding population. As the recurrent parent CML376 was also genotyped, it was possible to check if favorable alleles in our semi inbred experimental lines are derived from the CML376 or the landraces. The allele frequency of SNPs associated with the traits, and derived from the landraces, was calculated for the entire population, and stratified for each landrace family group. This allowed verifying if specific landrace is a potential donor for the allele of interest.

On the other hand, regarding selection, it is also important to know the distribution of the superior allele across the landrace progenies in the breeding population, regardless of its origin, landraces or the elite recurrent parent line. The superior allele is the one that causes a positive variation in the trait of interest. Therefore, we also calculated, for the significant SNPs, the frequency of the alleles with positive effects for each landrace progeny.

To evaluate the population in terms of favorable alleles content, the 20 families were ranked by the sum of the ranks of the superior allele frequency for each SNPs. Two rankings were made, one considering all significant SNPs, regardless of the origin of the superior allele, and the other considering only the markers where the superior allele is from landraces. Thus, with the former we assess which landraces could be more suitable for selection and with the latter, which landraces would be the best source of superior new alleles.

### GWAS Validation

Validation was performed by two approaches. In the first, we compared the results of GWAS over BC1S1 and BC1S2. In the second, we compare the BLUPs obtained from the TC2 yield trials with the genetic value estimative of BC1S2 individuals based on the effects of significant SNPs, obtained from BC1S1 GWAS. The genetic values estimative were calculated by multiplying the allele substitution effect vector by the marker matrix of BC1S2 individuals, as shown in the model below:

$${\hat{y}}_{2}={\text{Xα}}_{1}$$

where ${\hat{y}}_{2}$ is the vector (size n) of estimated genetic values in generation BC1S2, X is the genomic matrix of BC1S2 individuals, with dimension of n by m, where, n is equal to 174, that is, all the genotypes of BC1S2 generation, and m is the significant SNPs found in the BC1S1 GWAS, and finally, α_1_ is the vector of allele substitution value for each significant SNP. The prediction ability was measured by Pearson Product-Moment correlation $r_{\left({\overset{⌢}{y}}_{2},y_{2}\right)}$ where *y*_*2*_ is the vector of BLUPS of the genotypes in the TC2. This procedure was carried out to the tolerance index and grain yield in both irrigation treatments.

## Results

### Phenotypic Analysis

Based on the variograms surface trends, specific spatial factors were chosen as fixed effects to better fit the model to each site/irrigation treatment (Table 2). In all the individual trials, the Wald test confirmed the significance of using the spatial factors.

**TABLE 2:** Factors used in each model of first-stage spatial analysis (individual experiments).

Fixed spatial factors	Experiment (site/water management)
Row	SI/WW, SI/DR, TL/WW, LM/WW
Column	LM/DR
Row + Column	TL/DR

*Decisions about the use or not of spatial factors on the models were taken based on variogram plots.*

*LM, SI, and TL are, respectively, the locations Los Mochis, Santiago del Ixcutlia, and Tlaltizápan.*

*WW and DR are the irrigation treatments, well-watered, and drought conditions.*

Landraces progenies and the genetic check (Tester × CML376) distributions of grain yield means (Supplementary Figure 1) and the drought tolerance index (Supplementary Figure 2) show that there are landrace progeny genotypes performing better than the genetic check, which is a desirable situation for selection. Significant differences among genotypes were detected for grain yield under well-watered conditions (Table 3). Under drought and for the index, genetic differences were detected only to a 0.2 *p*-value threshold, both with a *p*-value of 0.15 (data not shown).

**TABLE 3:** Wald statistic for fixed effects and variance components for random effects to the joint analysis (second stage) for the three traits, grain yield under well-watered (GY WW) and drought (GY DR) and the drought tolerance index (HMRP).

Factor	GY WW		GY DR		HMRP	
**Fixed effects (Wald)**						
Location	364	***	201	***	8	*
**Random effects (LRT)**						
Genotype	0.03	**	0.03	+	0.0007	+
Genotype x Site	0.58	***	0.00	ns	0.03	***
Acc. (%)	19.9		25.5		20.1	
CVg	2.98		6.14		3.64	
CVe	20.76		32.85		25.13	
Heritability	0.04		0.17		0.06	

*The accuracy (Acc%), coefficients of genetic (CVg), and error (CVe) variation and heritability are also presented.*

ns, not significant; + *p* < 0.2; *p < 0.05; **p < 0.01; ***p < 0.001.

Overall, the heritabilities were low, considering the joint analysis for the drought-tolerance index, GY in drought (GYDR) and well-watered (GYWW) conditions, as expected for such complex traits (Table 3). The heritability of HRMP (0.06) and GYWW (0.04) were lower than for GYDR (0.17). The accuracy was similar for the three traits, with a higher value for GYDR (25.5%).

**FIGURE 2:**
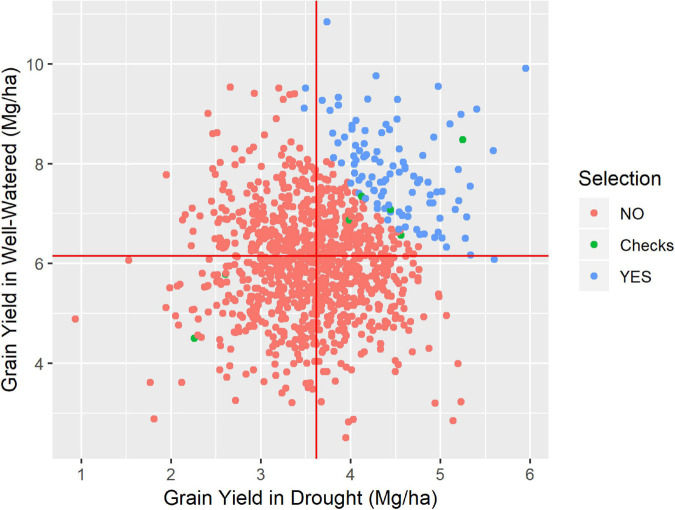
Grain yield at drought condition against the grain yield at well-watered condition, for the Los Mochis trial, where 1067 genotypes were tested. Highlighted in blue the 104 highest drought-tolerant, simulating a 10% of selection intensity, the six checks used (green) and the 956 not selected by the simulated selection (red).

The Harmonic Mean of the Relative Performance index measures drought tolerance. Its values are around 1.0, with higher values being more tolerant genotypes and lower values less tolerant. Regardless of site, the HMRP mean is always close to 1.0 because it is relative to the population in each site (Table 4). The genotypes with higher drought tolerance index values are also those that presented higher yield under both irrigation treatments (Figure 2). In BC1S1, the tolerance index to the location TL presents a higher variance than the other locations, and LM the lower. In contrast, in BC1S2 generation the LM presents a variance higher than TL and PV, and in general, the amplitude of the drought tolerance index was lower in BC1S2 than in BC1S1 (Supplementary Figure 3) what was expected as the genetic diversity was higher in the first generation, before applying selection.

**TABLE 4:** Minimum, mean, maximum, and variance of drought tolerance index for each site (LM, Los Mochis; TL, Taltizapan; and SI, Santiago del Ixcuintla) and overall sites for the generation BC1S1 and BC1S2.

	BC1S1				BC1S2			
	Min.	Mean	Max.	Var	Min.	Mean	Max.	Var
Overall	0.320	0.983	1.710	0.146	0.745	0,998	1.308	0.077
LM	0.389	0.984	1.627	0.143	0.546	0,994	1.459	0.141
TL	0.196	0.967	1.635	0.207	0.794	0,998	1.337	0.096
SI	0.303	0.966	1.551	0.198	−	−	−	−
PV	−	−	−	−	0.637	0.992	1.278	0.096

### GWAS

The use of principal components (PC) as covariates into the GWAS model was necessary as the population structure might result in a spurious marker-trait association. This necessity was confirmed by comparing the QQ-plots of the GWAS model with no PC as covariates and the QQ-plots of models containing it (QQ-plots not shown). The QQ-plots showed a better adjustment in the models that consider the correction for the first PCs. However, the sum of variance explained by the first three PCs was just 5.58%, distributed by 2.76, 1.44, and, 1.38%, evidence that the population was not very structured. Plotting the first two PCs no clear stratification in the population is noted Supplementary Figure 4), despite that the landraces families exist in the population. Only when considering the third PC is it possible to separate the group of landraces into SNLP166 and CHIH87 progenies. The lack of population structure gives one positive side to the analysis, since there is a lower risk of false-positive due to a possible structure, which is reduced further with the use of PCs as a covariate in the model.

**FIGURE 3:**
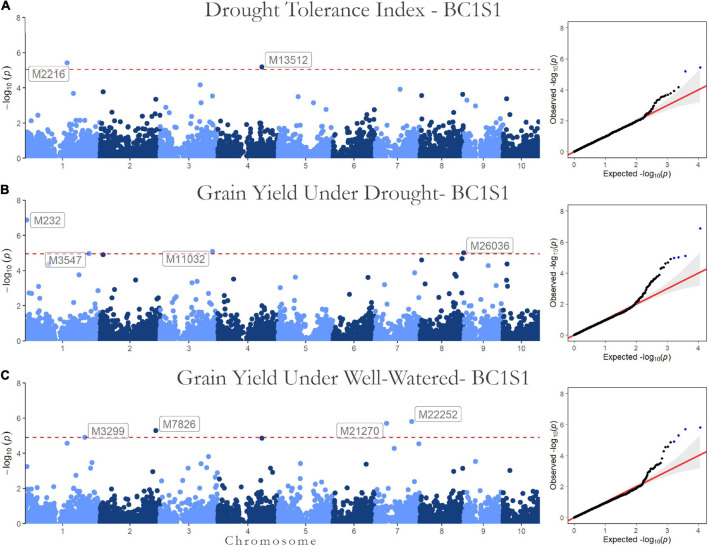
Genome-wide Association results for the first generation (BC1S1-TC1). Manhattan plot and QQ-plots for the **(A)** drought tolerance index, grain yield in **(B)** drought, and **(C)** well-watered conditions.

**FIGURE 4:**
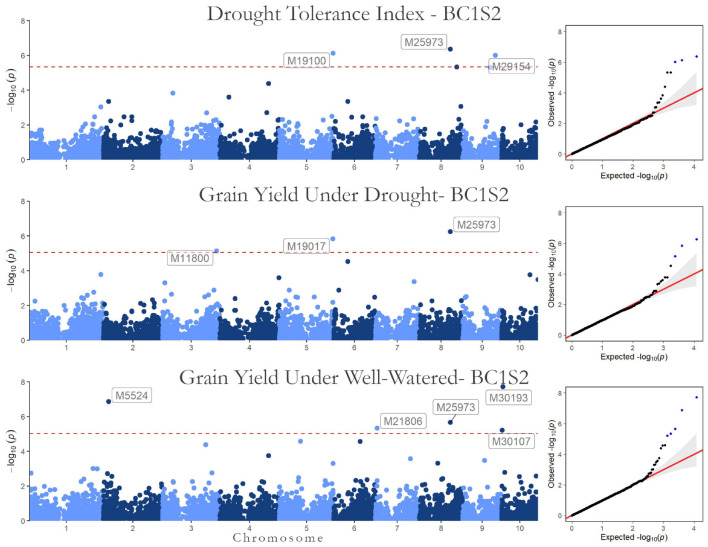
Genome-wide Association results for the second generation (BC1S2-TC2). Manhattan plot and QQ-plot for the drought tolerance index, grain yield in drought, and well-watered conditions.

The association results (*p*-values of the marker-trait association in a Manhattan plot and the quantile-quantile plots) are shown in Figure 3 for BC1S1 and in Figure 4 for BC1S2. The quantile-quantile plots (QQ-plot) is a plot of the negative log observed *p*-values against the distribution of expected *p*-values, i.e., under the null hypothesis, that there is no association between the SNP and the trait. This is a simple tool to check how well the chosen GWAS model absorbed the population structure (covariates in the model). The dots on the upper right section of the graph are the SNPs with some association with HMRP. We expected that most markers would not be associated with the trait. Looking at the QQ-plot, most markers have a *p*-value observed close to the expected null hypothesis, which can be verified by the line-shaped markers’ *p*-values that are aligned to the red line (observed equals the expected).

Regarding the BC1S1 results, the thresholds calculated from the permutation (8.78 × 10^–6^ for HMRP, 1.13 × 10^–5^ for GY in DR, and 1.28 × 10^–5^ in WW) were very close to the Bonferroni correction (0.05/*N* = 9.01 × 10^–6^) for the three GWAS procedures, considering the logarithmic transformation −*log*_10_. Two markers associated with HRMP were found (Figure 3A), one on chromosome 1 (M2216) and another on chromosome 4 (M13512). For drought treatment, four associated markers were found (Figure 3B), two on chromosome 1 (M232 and M3547), one on chromosome 3 (M11032), and the last on chromosome 8 (M26036). For the well-watered treatment, four markers were significant (Figure 3C), on chromosomes 1 (M3299), 2 (M7826), and a two on chromosome 7 (M21270 and M22252). The minor allele frequency of these 10 SNPs ranged from 5.20% (M26036) to 31.24% (M7826). The significant SNPs together explain just 0.04% of the drought tolerance index phenotypic variation (*r*^2^), and 0.07 and 0.08% of the grain yield phenotypic variation under drought and well-watered treatments (Table 5).

**TABLE 5:** Significant SNPs for drought-tolerance index and grain yield in drought and well-watered conditions, with the SNP position (chromosome and position), polymorphism (PM) of the SNP (Reference > Alternative), genotype of CML376 (CML) for the given SNP, minor frequency allele, effect of allele substitution, heritability, and *r*^2^ for the GWAS of BC1S1 generation.

Trait	SNP	Chr	Position	PM	CML	MAF	Effect	*h* ^2^	*r* ^2^
HMRP	M2216	1	171,883,409	G > C	GG	7.31%	−0.020	0.014	0.00020
	M13512	4	180,305,244	C > A	AA	9.37%	0.018	0.014	0.00019
GY/DR	M232	1	7,760,066	A > G	AA	6.47%	−0.059	0.017	0.00027
	M3547	1	261,017,573	A > G	AA	22.12%	0.035	0.016	0.00026
	M11032	3	214,051,229	G > A	GG	8.00%	0.040	0.009	0.00087
	M26036	8	178,731,659	A > T	AA	5.20%	0.052	0.011	0.00011
GY/WW	M3299	1	243,650,161	G > C	GG	10.52%	0.052	0.009	0.00009
	M7826	2	227,370,542	G > T	TT	31.24%	0.051	0.021	0.00044
	M21270	7	46,333,211	A > G	AA	7.31%	−0.064	0.011	0.00011
	M22252	7	149,023,564	G > T	TT	5.55%	0.084	0.013	0.00019

**FIGURE 5:**
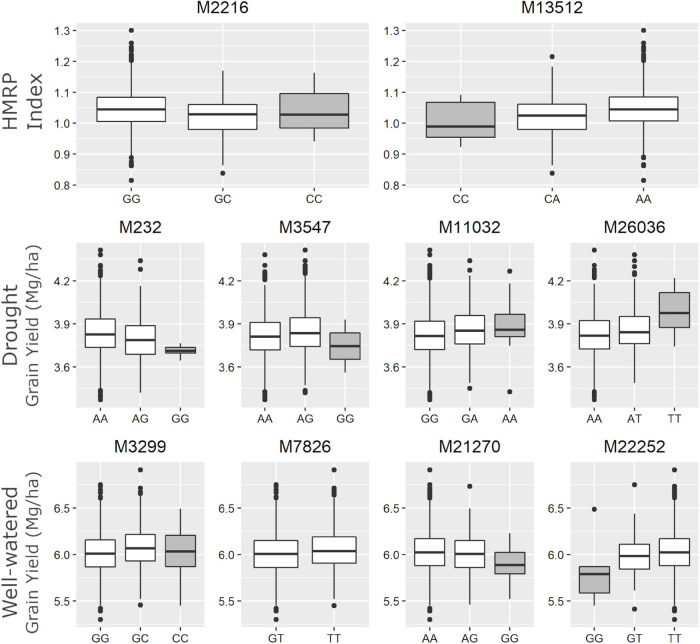
Boxplot of drought tolerance index (first row - HMRP) and grain yield in drought and well-watered conditions (second row – GYDR, third row - GYWW) of each genotype class for the 10 associated SNPs. The gray boxes highlight the homozygote genotype for the landrace origin allele.

The SNP M7826, associated with GYWW, was the only marker (among the ten) that did not have any individual in one of the homozygous classes (Figure 5). The alternative homozygous at M13512 showed a slight increase in the HMRP. In general, the heterozygous class tended to be between the homozygous classes, following a trend of additive substitution effect, except to the SNPs M3547 and M3299, where the heterozygous class had a higher grain yield (in DR and WW, respectively) than the homozygous classes. The SNPs M26036 and M11032, associated with GYDR, suggest a higher GY when homozygous for the alternative allele. Conversely, the SNPs M232 and M3547 showed lower yield when homozygous for the alternative allele. When homozygous for the alternative allele, the SNP M21270 is less productive in well-watered treatments. In contrast, the SNP M22252 shows an opposite direction, higher yields when homozygous to the alternative.

### Validation of Significant Markers Over Irrigation Treatments and Generations

The significant markers found using in this research were not stable across traits in the first generation (BC1S1). The two SNPs associated with the drought tolerance index were not associated with grain yield. Likewise, none of the SNPs associated with grain yield were in both irrigation treatments. Furthermore, none of the 10 SNPs found by GWAS in BC1S1-TC1 (Table 5) were significant in the BC1S2-TC2 GWAS (Table 6 and Figure 4). The BC1S2-TC2 GWAS had assigned 3 SNPs for drought tolerance index and 3 for grain yield under drought treatments and 5 for grain yield in well-watered treatments. In the second generation, one SNP was stable across the traits. The SNP M25973 was associated with the drought tolerance index and with the grain yield in both irrigation treatments, although it was not detected in the first generation.

**TABLE 6:** Significant SNPs for drought-tolerance index and grain yield in drought and well-watered conditions, with the SNP position (chromosome and position), minor frequency allele, effect, heritability, and *r*^2^ for the GWAS of BC1S2 generation.

Trait	SNP	Chr	Position	MAF	Effect	*h* ^2^	*r* ^2^
HMRP	M19100	5	221,581,023	7.35%	−0.028	0.039	0.0015
	M25973	8	128,738,707	7.06%	0.030	0.042	0.0018
	M29154	9	133,967,900	6.47%	−0.031	0.041	0.0017
GY/DR	M11800	3	222,428,531	7.06%	−0.137	0.027	0.0007
	M19017	5	220,204,698	7.94%	0.155	0.039	0.0015
	M25973	8	128,738,707	7.06%	0.188	0.051	0.0026
GY/WW	M5524	2	21,392,902	11.76%	0.350	0.056	0.0031
	M21806	7	7,471,998	5.88%	0.358	0.031	0.0010
	M25973	8	128,738,707	7.06%	0.366	0.038	0.0015
	M30107	10	3,024,706	14.71%	−0.216	0.026	0.0007
	M30193	10	5,754,305	5.59%	−0.485	0.054	0.0029

The correlation between the predicted values of the BC1S2 estimates based on the effects of allelic substitution of significant SNPs obtained from BC1S1 GWAS, and the observed BLUPs was –0.11 for the drought tolerance index, –0.20 for grain yield under drought treatments and –0.23 under well-watered.

### Gene Annotation and Allele Origin

**FIGURE 6:**
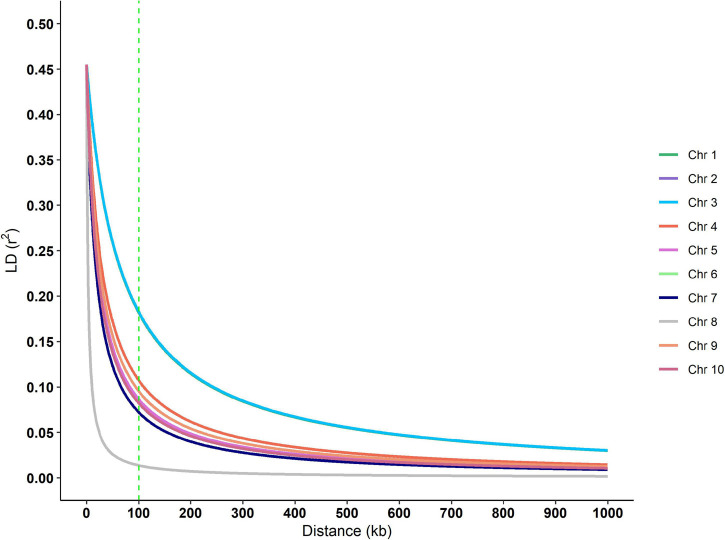
Linkage disequilibrium decay over each chromosome of the BC1S1 population, that is, the breeding population used in this study originated from 20 landraces that undergone through a backcross with an elite line and selfed. The vertical dashed green line indicates the window size used in the annotation process.

Putative genes for drought tolerance were identified by searching the B73 genome in the regions around the ten significant SNPs identified in the analysis (Table 7). The search was performed inside a 100k bp window up and down-stream of the markers. The linkage disequilibrium (LD) decay estimative from the BC1S1 data can be observed by plotting the *r*^2^ against the distance of base pairs (Figure 6), a rapid decay occurs while increasing the chromosome distance between two loci for all the chromosomes. The overall LD decay extent was in a range of 50–300k bp and just for the chromosome number 3 the *r*^2^ does not decrease to less than 0.13 within 100k bp. A more abrupt decrease of the LD was observed in chromosome 8 and, in contrast, a smoother decay was observed in chromosome 3. All the other chromosomes presented a similar decay pattern.

**TABLE 7:** Description of SNPs, their positions, number of genes (putative and described) found in a search window of 100k bp up and downstream from the mark positions.

SNP	Chr	Position	Number of finds	Most described promising finds	Protein type/family
M2216	1	171.883.409	1	Zm00001d030982	Ankyrin repeat family protein
M13512	4	180.305.244	9	Zm00001d052123	Thioredoxin-like 5
M232	1	7.760.066	4	Zm00001d027539	Glutathione transferase 1^*^
M3547	1	261.017.573	6	Zm00001d033375	Golgi-body localization protein domain
M11032	3	214.051.229	9	Zm00001d043929	Fasciclin-like arabinogalactan^*^
M26036	8	178.731.659	14	Zm00001d012693	Transmembrane transport protein
				Zm00001d012699	Crystal structure of auxin-binding protein 1
M3299	1	243.650.161	3	Zm00001d032931	Oxoglutarate/iron-dependent dioxygenase
M7826	2	227.370.542	8	Zm00001d007301	Protein disulfide isomerase
				Zm00001d007307	Magnesium transporter NIPA4
M21270	7	46.333.211	20	Zm00001d019612	Protein transport protein SEC31
M22252	7	149.023.564	6	Zm00001d032932	Agmatine coumaroyltransferase
				Zm00001d021332	Peptidyl-prolyl *cis*-*trans* isomerase CYP21

*The most described genes founded in each search and the protein type/family that these most described genes encode.*

^*^ Finds described in the discussion and detailed in Table 8.

The SNP M2216 is 60k bp upstream from a predicted gene that is still not characterized. M13512 is in a region of chromosome 4 that has nine putative protein coding sequences. The closest gene to M232 SNP is located 85k bp upstream from it, and other three putative genes are less than 60k bp away. M3547 SNP is in an intron of a putative gene and there are five more probable genes inside its search window. M11032 is also in an intron of an uncharacterized putative gene, with eight more putative genes around it. The SNP M26036 is in a region with 14 possible genes. M3299 is in an exon of an uncharacterized predicted gene and with two more in its search window. M7826 is 1.5k bp only from a putative gene, and in less than 50k bp there are seven more predicted genes. The SNP M21270 is on a chromosome region with 20 putative genes. Moreover, in the M22252 window, we could find six predicted genes. Clearly, a window of 100k bp can host a large number of genes, and possibly many more than those listed here, as annotation and gene discovery is a work in process.

Regarding the allele origin, the frequency of landrace alleles in the BC1S1 lines ranges from 5 to 31%. The SNPs M3547 and M7826 present the highest frequencies for the landrace allele among the ten significant SNPs (Supplementary Figure 5). The favorable allele for four SNPs (M3547, M11032, M26036, and M3299) came from the landraces. Populations originating from the NVOL46, COAH20, COAH21, CHIH87, and COAH78 landraces are those with a higher frequency of the superior allele for these 4 SNPs (Landrace origin rank on Table 1 and Supplementary Figure 5). Regardless of the origin of the superior allele, the five landrace populations that present a higher frequency of superior allele were those obtained from SNLP17, NVOL46, COAH78 CHIH85, and COAH20 (general rank on Table 1 and Supplementary Figure 6). This suggests that these populations have a higher frequency of favorable alleles, being a good source for drought tolerance.

## Discussion

### Field Trials and Phenotypes

All the field trials presented strong spatial (row and columns) trends. Therefore, the spatial modeling used in the first stage analysis was crucial to avoid, or minimize, their effects on the means, mainly in the WW treatments, where augmented design was carried out. Thus, we expected lower heritabilities in these trials (WW) than in the DR treatments, even though drought-stressed trials usually are more affected by field spatial trends (Badu-Apraku et al., 2004), resulting in lower heritability for yield. That was one of the reasons we chose to use two repetitions in the DR treatments, to be able to estimate better the residual variance and to avoid the possible complete loss of genotypes, since under stress the number of missing plots is often higher, which would greatly affect the genetic estimates in case there is only one plot of each genotype. Another reason that would explain the greater heritability in drought than in the irrigated condition may be the fact that a diverse population when tested in a stressful environment may have its individuals better discriminated than if tested in a stress-free environment, that is, genetic diversity may be more evident in the phenotypic response when in a stressful environment, which may be reflected in heritability.

As the TC1 used a single tester, the tester by genotype interaction could not be separated from the estimated genotypic value (BLUEs and BLUPs). Thus, the first generation (BC1S1) data are biased by this interaction, which is acceptable since it is not feasible to perform a testcross trial with more than 1,000 genotypes crossed to a group of testers. In the second testing year this was minimized, as with only 174 BC1S2 lines, testcrossing with three testers was possible.

Similar values are found when comparing the grain yield means and the drought tolerance index for the BC1 progeny testcrosses and the genetic checks (Tester crossed with CML376). However, the distribution suggests a superior performance for the BC1 progenies for grain yield (Supplementary Figure 1), and for the drought tolerance index (Supplementary Figure 2). It can be inferred that there are novel alleles originating from the landrace donor parent that contribute to drought tolerance and/or a positive interaction between genes from the different sources, landraces or CML.

The usage of HMRP and other index based on harmonic mean is recognized for stress tolerance studies (Jafari et al., 2009; de Mendonça et al., 2017; Lyra et al., 2017). The Harmonic Mean Relative Performance was confirmed as a simple and efficient index that allows the selection of lines that perform well in both the DR and WW irrigation treatments, achieving the objective of these “stressed x not stressed” trials (Figure 2). Using the HMRP index, the breeder can select the highest performing lines in both treatments or those that perform very well in one and close to the average in the another. Additionally, the breeder can avoid selecting those that underperform in at least one of the treatments. Sites and line by site interaction effects were significant regarding the index (Table 3). Therefore, even if the means across the sites are similar, differential expression of the drought tolerance was identified, and selection based on results by location should be avoided.

### Genotyping

**FIGURE 7:**
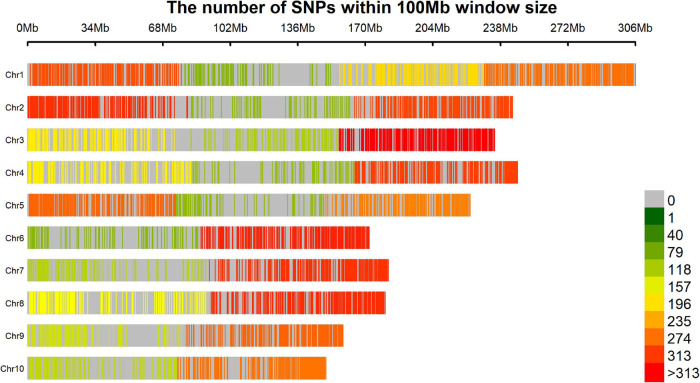
Result of coverage genome showing the SNP density within a 100 Mb window.

We believe that the final number of SNPs (5,695) was relatively low considering the start point of 47,074 markers comparing with others maize GWAS published (Xiao et al., 2017), mainly because the strict quality control filters in terms of missing data, however, the markers were widely distributed across the genome, covering all chromosome arms without any large uncovered segment and the low density areas are usually around the centromere region (Figure 7). This good genome coverage is important for this kind of study and considering the good quality of the final set of 5,695 SNPs due the strict quality control process, the relative low number is not a limitation. This number of markers may be due to the genetic mating design of the population. Crossing and backcrossing to a homozygous line (CML376) could dilute allele frequency. It could also be a reason for the restricted population structure (estimated by the PCA), even though the lines originated from 20 different landrace parents, and for the low minor allele frequency (MAF) of the SNPs associated to the traits (Table 5). Another important detail that should be considered is that the germplasm of the reference genome used to obtain markers positions (B73 RefGen_v4) has a temperate origin and certainly presents incongruities with landrace derived materials, however, it is the best reference genome available at that moment. It is important to remember that maize landraces, due to heavy transposon activity and other factors, often contain large amounts of insertions, deletions, and reorganized chromosomes. Perhaps, if a closer genome had been used to position the markers, we may have an even better coverage, however, because our population derived from Landraces crosses would be difficult to get any closer reference genome. The drastic reduction on the final set of markers, from 32,592 to 5,695, occurred during the quality control process; the call rate used was high (95%) to ensure a high-quality set of markers over a large set with many imputations.

### GWAS

Many researchers use correlated traits to study drought tolerance such as seedling survival rate under drought (Mao et al., 2015), ear length and kernel number per row (Lu et al., 2006), and one of the most used, Anthesis-Silk Interval (ASI) (Yu, 1994; Bolaños and Edmeades, 1996; Vargas et al., 2006; Wang and Qin, 2017). This is due to the low heritability of grain yield compared with the correlated traits. Xue et al. (2013) using a diversity panel of CIMMYT lines and studying nine traits related to drought tolerance found 42 SNPs associated with those traits, any one of the SNPs listed by the authors was detected by our study as significant for the HRMP index. Setter et al. (2011) in an association mapping study of abscisic acid levels during drought stress in maize found 8 polymorphisms related with drought tolerance. One of those polymorphisms was used for the annotation of the maize gene Zm00001d034385, a validated gene associated with drought tolerance. It is mapped 24M bp from the M3547 SNP in the present study that was associated with GYDR. Although it is on the same chromosome arm (chromosome 1, long arm), it is a considerable long distance away, given a LD decay of ∼100k bp.

The LD decay reported in maize varies considerably among populations (Flint-Garcia et al., 2003). In landraces or a highly diverse panel, the LD decay is reported to be smaller than in elite inbred lines, by a magnitude of 1k bp for landraces and ∼500k bp for elite inbred lines (Yan et al., 2009). Additionally, the average LD decay distance in tropical germplasm (∼10k bp) is shorter than in temperate germplasm (∼100k bp) (Lu et al., 2011). The populations used in this study were at the midpoint as the initial subtropical landraces were the donor parent in a backcross where an elite inbred line was the recurrent parent.

We focused our search for genes in a window of 100k bp around the 10 trait-associated SNPs. The LD extent for our population (50–300k bp) is in accordance with those observed in the literature. This is the expectation for material that are neither landrace nor elite inbred line, since the former normally has a shorter LD decay than evidenced in our experimental lines and the latter, a larger LD decay (Remington et al., 2001; Flint-Garcia et al., 2003). Except for two cases, our genome search found putative genes that are not completely described. These two cases are SNP M232 that is associated with GYDR and is located 85k bp close to the gene; and Zm00001d027539 that belongs to the glutathione S-transferase (GST) gene family (GST, EC 2.5.1.18). This second gene family plays an important role in plant response to environmental conditions (McGonigle et al., 2002) plus some abiotic stresses such as herbicide (Cataneo et al., 2003), heat (Cetinkaya et al., 2014), and drought (Xu et al., 2015; Min et al., 2016). In Arabidopsis, plants with mutated GST gene were more tolerant to drought stress than wild-type plants (Chen et al., 2012). Additionally, there is the predicted gene Zm00001d043929 associated with GYDR in the present study and mapped to ∼80k bp upstream from the M11032 SNP. This gene encodes a protein with a fasciclin-like domain (FAS1), and this domain is found in Fasciclin-like arabinogalactan (FLAs) proteins that are involved in cell adhesion. Also, some FLAs have their expression regulated by the phytohormone abscisic acid (ABA) (Johnson et al., 2003). Both FLAs and ABA play an important role in plant response to stress (Faik et al., 2006; Finkelstein, 2013; Zang et al., 2015). Wang et al. (2016) points out that the participation of FLAs in enhancing cell wall synthesis in response to drought stress, which could minimize water loss and cell dehydration. Reduction of some FLA gene expressions is also associated with kernel abortion in maize (Cagnola et al., 2018) leading to a reduction in kernel number and thus lowering yield.

These two findings (Table 8) are promising for further maize breeding studies. In our experimental lines, we detected markers within ∼80k bp of these genes. Thus, fine-mapping and allele variant studies in these specific *loci* could help to elucidate their role and could be included in a genomic selection set. These two markers (M232 and M11032) could be used in our experimental lines to drive the selection in the next generations and be a basis for further investigations. This is especially so for the M11032 SNP, where the alternative allele originates from the landrace (Figure 5) is the superior one, and the homozygous state for this allele had a slightly higher grain yield in drought treatments. The landraces ARZM12193, CHIH338, CHIH87, COAH19 are not always good sources for the superior allele, since the frequency of M11032 is null in these populations.

**TABLE 8:** Two significative SNPs located close to putative genes that encode proteins associated with plant response to drought.

SNP	Distance	Putative gene and protein type	References
M232	84.3k bp	Zm00001d027539 Glutathione S-transferase	McGonigle et al., 2002; Chen et al., 2012; Xu et al., 2015; Min et al., 2016
M11032	80.7k bp	Zm00001d043929 Fasciclin-like arabinogalactan	Johnson et al., 2003; Faik et al., 2006; Finkelstein, 2013; Zang et al., 2015; Wang et al., 2016; Cagnola et al., 2018

*The distances between the mark and the putative gene in pair of bases, the putative gene name, the protein encoded type/family and references that studied these proteins with drought stress in plants.*

There were three more significant SNPs (M3547, M26036, and M3299) where the superior allele is from the landraces, which demonstrates new diversity for drought tolerance alleles in the experimental lines. In this context, the landraces NVOL46, COAH20, COAH21, CHIH87, and COAH78 would be the main sources for exploring this diversity since they have a higher frequency of these favorable alleles.

### Validation and Significant Markers Over Irrigation Treatment and Generations

For the BC1S1 testcrosses, the lack of significant SNP correlation among the irrigation treatments may be a sign of differential expression of genes under contrasting water environments. For example, Cagnola et al. (2018) demonstrated that drought stress could induce a reduction in FLA gene expressions, which may cause kernel abortion in maize resulting in reduced grain yield. As discussed above, there is a putative gene inside the SNP M11032 window that belongs to this gene family.

Likewise, there was no correlation of significant SNPs between the BC1S1 and BC1S2 generations. This is to be expected since selection was made in BC1S1 to generate the BC1S2 we induced a non-random allele frequency change between the generations that can affect the results as the new generation is diverging from the first (Henshall, 2013). Thus, in our study, the use of the BC1S2 generation to validate the GWAS results of BC1S1 generation was not effective.

Additionally, the use of allelic substitution effects estimated using the BC1S1 data to predict the phenotype of BC1S2 generation as a validation method was not successful. The correlations between the predicted values and the observed BLUPs were low and negative to the three cases (–0.11 for the drought tolerance index, –0.20 for GYDR treatments and –0.23 for GYWW). The allele frequency also interferes with the average effect of the allele. Therefore, the selection and its incumbent frequency allele changes, also results in allelic effect deviations estimated in BC1S1, which could not be constant through generations of selection. Moreover, the heritabilities were low in BC1S1 (0.04–0.17), and the significant SNPs explain less than 0.1% of phenotypic variation, that is indicative of the complexity of quantitative traits as drought tolerance and grain yield, that reduces the efficiency of using a few SNPs for prediction, even if they are significant. Finally, there is the effect of the tester on the BC1S1 phenotypic data since the interaction tester by landrace progenies could not be estimated in this case. Thus, the allelic substitution effect is biased for this specific testcross and to use it to predict the genotypic values of a second testcross involving another tester may be unfeasible.

Early testcross is a strategy to take advantage of early selection for yield and other traits that avoids excessive resource spending on unpromising families, and it can also be applied in a pre-breeding program. However, as Bernardo (2010) concludes, the low heritability limits its effectiveness. The GWAS in early generations achieved promising results and was demonstrated to be a useful tool for identifying new genetic variants in our landrace population. Nevertheless, a practical and effective method to validate a GWAS in this situation still needs to be developed.

### Favorable Alleles and Their Origin

The frequency of allele from landrace over the entire population are the same values of the MAF (Table 5), once the allele from landraces is, as expected, the allele in minor frequency. Landrace origin rank (Table 1 and Supplementary Figure 5) gives the idea of which landraces are the most promising source for positive variants, and it suggests that the landraces NVOL46, COAH20, COAH21, CHIH87, and COAH78 are those that could be used for further investigations regarding new variants, at the same time, the populations originated from them could be more explored by breeders to exploit this new variant superiority.

The general rank (Table 1 and Supplementary Figure 6) gives the idea of which landrace populations are the promising to selection regards the combination with adapted elite genitor. This suggests that the SNLP17, NVOL46, COAH78 CHIH85, and COAH20 populations have a higher frequency of favorable alleles and/or their combination, being a good source for drought tolerance.

## Conclusion

The GWAS analysis was able to identify genomic regions associated with drought tolerance in early generation testcrosses, although they were not stable over generations or irrigation treatments. Two promising putative genes that encode proteins related with the physiological plant response to abiotic stress are located close to the significant SNPs found in this study. Some alleles from the landraces provide a slightly higher yield under drought treatments. These results indicate that the diversity delivered from landraces x elite inbred line backcrosses can play an important role for improving the maize tolerance to drought, and for trait introgression bringing new superior allelic diversity from landraces to breeding populations. As we expected for a complex trait such as drought tolerance, we did not find a unique marker with a large genetic effect, but a handful of regions that can be transferred to advanced and adapted breeding populations using landraces as origin of positive variants.

## Data Availability Statement

Publicly available datasets were analyzed in this study. This data can be found here: dataverse: https://hdl.handle.net/11529/10548540.

## Author Contributions

PB wrote the manuscript, analyzed the genetic and field data, prepared the genetic markers data, and participated in the conceptual design of the research. TM and MW created the conception of the study. TM and MA designed and carried out the experiments. VV-M and ES-I were responsible for the field operations. TM, RF-N, KS, and GG reviewed the manuscript. JB supported and reviewed the statistical and spatial analysis. CP conducted the genotyping process in the laboratory and the first marker data preparation. All authors contributed to the article and approved the submitted version.

## Conflict of Interest

The authors declare that the research was conducted in the absence of any commercial or financial relationships that could be construed as a potential conflict of interest.

## Publisher’s Note

All claims expressed in this article are solely those of the authors and do not necessarily represent those of their affiliated organizations, or those of the publisher, the editors and the reviewers. Any product that may be evaluated in this article, or claim that may be made by its manufacturer, is not guaranteed or endorsed by the publisher.

## Footnotes

1
http://www.diversityarrays.com

2
https://www.diversityarrays.com/

3
www.ncbi.nlm.nih.gov
