# Supplementary material for: Introgression of Maize Diversity for Drought Tolerance: Subtropical Maize Landraces as Source of New Positive Variants
Source: Front Plant Sci. 2021 Sep 23;12:691211. doi: 10.3389/fpls.2021.691211 (PMC8495256; doi:10.3389/fpls.2021.691211)
Supplement: Supplementary file 1 [file Data_Sheet_1.zip › R codes.pdf]

Supplementary material – R codes

This supplementary material is part of the scientific article *Introgression of maize diversity for drought tolerance: subtropical maize landraces as source of new positive variants.* Frontiers in Plant Science, 2021.

The R code below describes the methodology of two-stage analysis used in this research

```
# The lines that start with # should not be run, they are comment or title lines

# loading asreml library
library(asreml) # asreml version 3 was used, be aware that the syntax changed if you use v.4
library(dplyr)

## loading the data

load("rawData_Pheno.RData") # Reading the file that contains the phenotypic data

## Data for each trial

LMWW <- df[df$trial=="LMWW" & df$generation== "BC1S1",] %>% droplevels # Los Monchis Well-Watered
LMDR <- df[df$trial=="LMDR" & df$generation== "BC1S1",] %>% droplevels # Los Monchis Drought
TLWW <- df[df$trial=="TLWW" & df$generation== "BC1S1",] %>% droplevels # Tlalti Well-Watered
TLDR <- df[df$trial=="TLDR" & df$generation== "BC1S1",] %>% droplevels # Tlalti Drought
SIWW <- df[df$trial=="SIWW" & df$generation== "BC1S1",] %>% droplevels # Santiago Ixcuintla Well-Watered
SIDR <- df[df$trial=="SIDR" & df$generation== "BC1S1",] %>% droplevels # Santiago Ixcuintla Well-Watered

## First stage - Trail-by-Trial (site-by-site)

# Running the single trial model for each trial (Trial = combination Location x Irrigation Treatment)

singleTrial <- asreml(fixed = GrainYield ~ 1 +
                      entry +
                      #R + C + # First we tried without this line and check the variogram, if any trend is
present we've added the dimension as factor (Row, Column, both or a combination of them, depending on the
trend)
                      Rep,# The DR trials had blocks. The WW did not, so, for WW trials we removed this
factor
                     rcov = ~ar1(R):ar1(C), # if there is any subset in the field we used
at(subset):ar1(R):ar1(C), to compute the correlation within each field subsection
                     data = dataSingleTrial) # replace the dataSingleTrial with the Trial to be analyzed
(LMWW,LMDR,TLWW,TLDR,SIWW or SIDR)

singleLoc <- update(singleLoc) # Use this to update the model if it does not get converged
plot(variogram(singleLoc)) # to check the variogram

blue <- predict(singleLoc,classify="entry")$predictions$pvals
blue$weigths <- blue$standard.error^(-2) # weights: The inverse of the variance of each blue (adjust
mean); higher the variance lower the weight.
blue$location <- dataSingleTrial$location[1]

# save each blue in its own R object. Example:
blueLMDR <- blue # if you have replaced the dataSingleTrial by LMDR
blueLMWW <- blue # if you have replaced the dataSingleTrial by LMWW
#and so on...

## Second stage - Combined analysis by Locations (the irrigation treatments are analyzed separately)

# combining the blues of the three locations in one data frame for DR and other for WW
blueDR <- rbind(blueLMDR,blueSIDR,blueTLDR)
blueWW <- rbind(blueLMWW,blueSIWW,blueTLWW)

# Analysis mode for Well-watered
```

```r
WW.joint <- asreml(fixed = predicted.value ~ Loc, # The trait now is the blue from the first stage
                   random = ~ Entry + Loc:Entry,
                   weights = weigths,
                   workspace = 1.6e+08,
                   pworkspace = 1.6e+08,
                   maxiter = 200,
                   data = blueWW)

# Variance components - WW
summary(WW.joint)$varcomp

VgWW <- summary(WW.joint)$varcomp[1,2]
VgeWW <- summary(WW.joint)$varcomp[2,2]
VeWW <- summary(WW.joint)$varcomp[3,2]

# CV and accurancy - WW
(CVgWW = (sqrt(VgWW)/(mean(blueWW$bWW)))*100)
(CVeWW = (sqrt(VeWW)/(mean(blueWW$bWW)))*100)
(acWW = (1-(1/(1+(2*((VgWW/VeWW)^1)))))^0.5)

# Heritability - WW
(H2.WW <- VgWW/
    (VgWW +VgeWW/3 + # 3 locations
       VeWW/3)) # 3 location, two reps per location

# Analysis model for Drought
DR.joint <- asreml(fixed = predicted.value ~ Loc, # The trait now is the blue from the first stage
                   random = ~ Entry + Loc:Entry,
                   weights = weigths,
                   workspace = 1.6e+08,
                   pworkspace = 1.6e+08,
                   maxiter = 200,
                   data = blueDR)


# Variance components
summary(DR.joint)$varcomp

VgDR <- summary(DR.joint)$varcomp[1,2]
VgeDR <- summary(DR.joint)$varcomp[2,2]
VeDR <- summary(DR.joint)$varcomp[3,2]

# CV and accurancy
(CVgDR=(sqrt(VgDR)/(mean(blueDR$bDR)))*100)
(CVeDR=(sqrt(VeDR)/(mean(blueDR$bDR)))*100)
(acDR=(1-(1/(1+(2*((VgDR/VeDR)^1)))))^0.5)

# Heritability - DR
(H2.DR <- VgDR/
    (VgDR + VgeDR/3 + # 3 locations
       VeDR/(3*2))) # 3 location, two reps per location
```

The R code below describes the methodology used to execute GWAS using FARMCPU R package

```r
###################### #
#### GWAS - FARMCPU ####
###################### #

#### FARMCPU e GAPIT Functions ### #
library(MASS) # required for ginv
library(multtest)
library(gplots)
library(compiler) #required for cmpfun
library(scatterplot3d)
library(bigmemory)
library(biganalytics)
library(compiler)
library(LDheatmap)
```

```r
library(genetics)
library(ape)
library(EMMREML)
source("http://zzlab.net/GAPIT/gapit_functions.txt")
source("http://zzlab.net/FarmCPU/FarmCPU_functions.txt")

########################### #
### GWAS Procedure BC1S1 ####
########################### #

### Organizing the data for GWAS
# Marks and map from snpReady
load("Geno_Data.RData")

# Using snpReady to get the Ga and PCA#
library(snpReady)
Ga <- G.matrix(M[,-1], method = "VanRaden", format = "wide", plot = F)$Ga
eig.result <- eigen(Ga)
lambda <- eig.result$values
PCA <- eig.result$vector

p1 <- FarmCPU.P.Threshold(Y = Y1,
                          GD = M,
                          GM = map,
                          trait = "GrainYield",
                          theRep = 300)

FARMCPU <- FarmCPU(Y  = Y1,
                   GD = M,
                   GM = map,
                   CV = PCA[,1:3],
                   method.bin="optimum",
                   maxLoop=10,
                   MAF.calculate = TRUE,
                   maf.threshold=0.05,
                   p.threshold = p1,
                   threshold.output = p1,
                   file.output = F,
                   cutOff = c(p1*(ncol(M)-1), 0.05))

results_GWAS <- FARMCPU$GWAS
```
