# Supplementary material for: Introgression of Maize Diversity for Drought Tolerance: Subtropical Maize Landraces as Source of New Positive Variants
Source: Front Plant Sci. 2021 Sep 23;12:691211. doi: 10.3389/fpls.2021.691211 (PMC8495256; doi:10.3389/fpls.2021.691211)
Supplement: Supplementary file 1 [file Data_Sheet_1.zip › Supplementary Figures and Tables.docx]

Supplementary Material

# Supplementary Figures


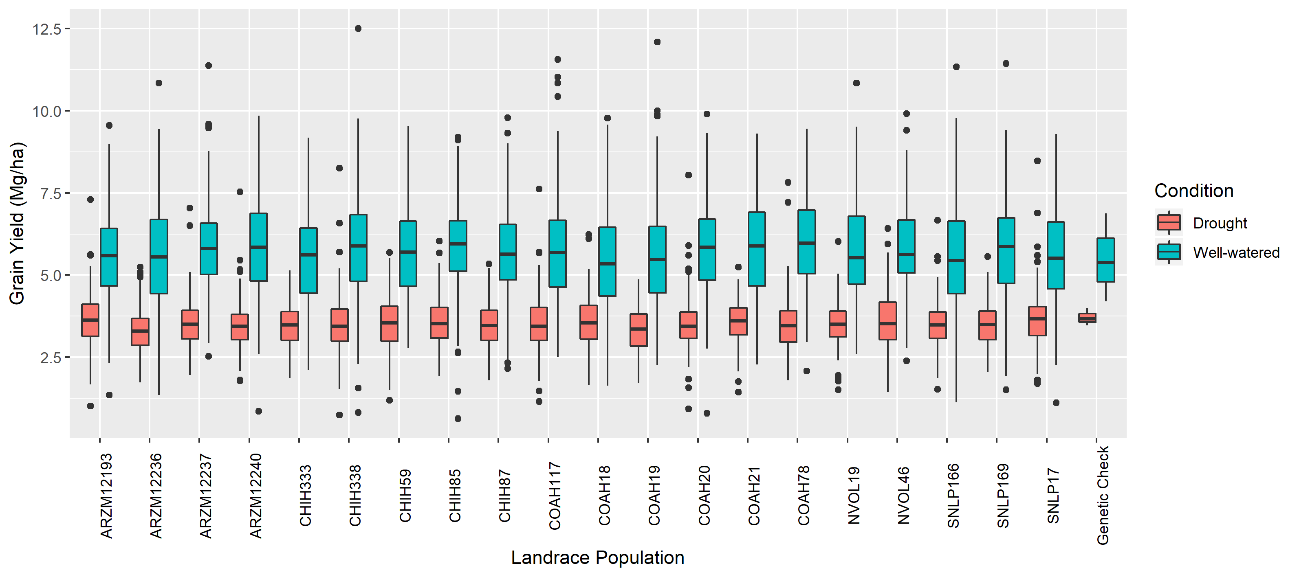


Supplementary Figure 1. Boxplot of landraces progenies and genetic check (Tester x CML376) for grain yield (Mg.ha^-1^) at both irrigation treatments.


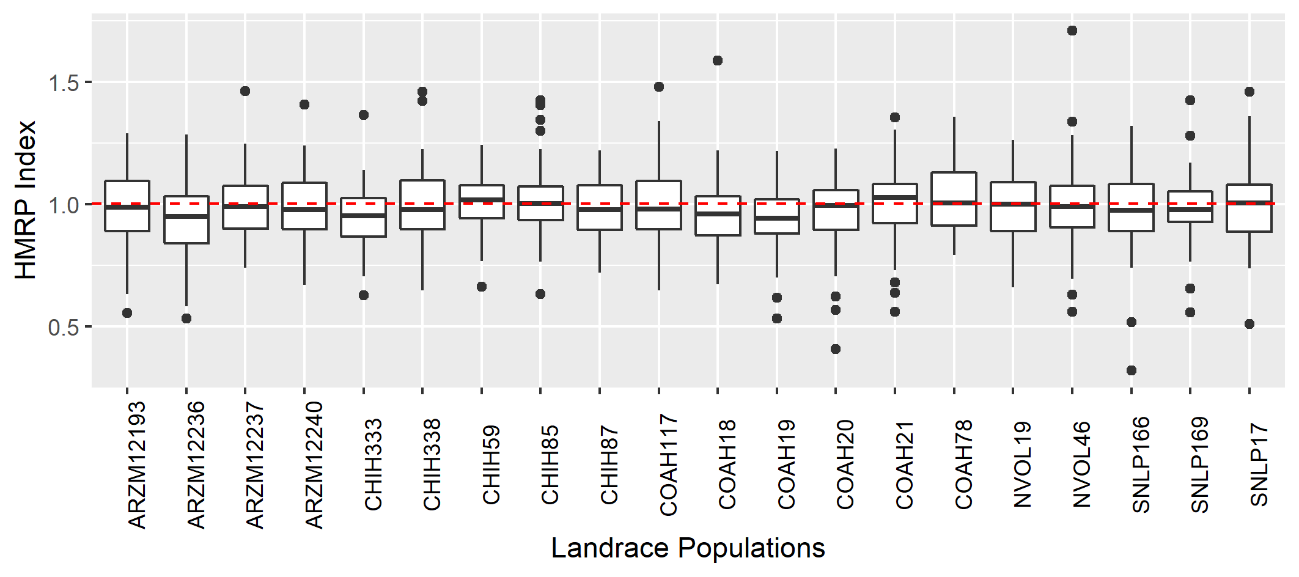
Supplementary Figure 2. Boxplot of landraces progenies and genetic check (Tester x CML376) for drought-tolerance index (HMRP) at both irrigation treatments. The red dashed line is the global mean HMRP of the genetic check (1.002616).


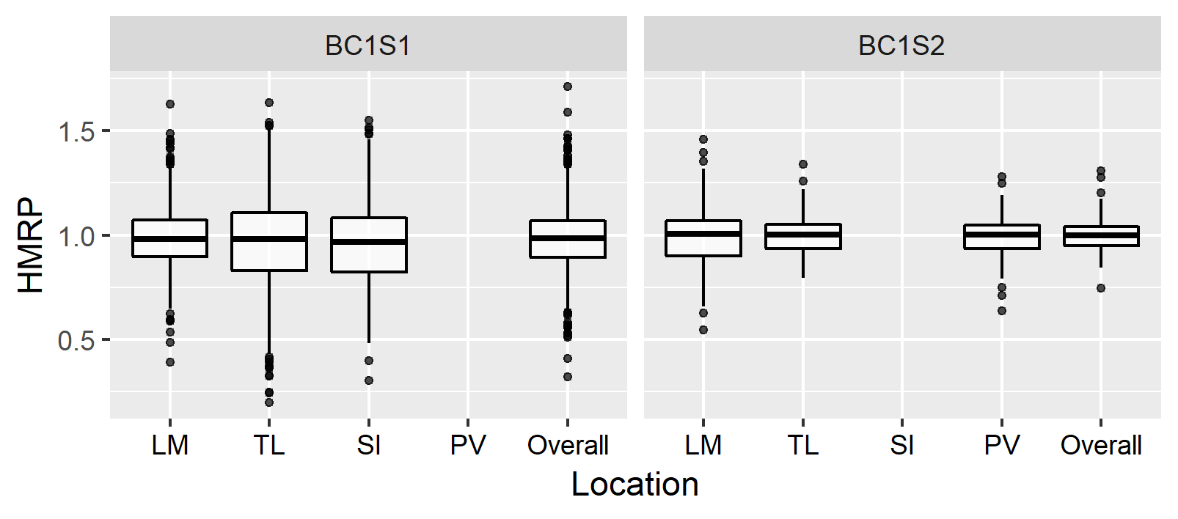


Supplementary Figure 3. Boxplot of drought tolerance index for each site (LM, Los Mochis; TL, Taltizapan and SI, Santiago del Ixcuintla, PV, Puerto Vallarta) and overall sites to the generations BC1S1 and BC1S2.


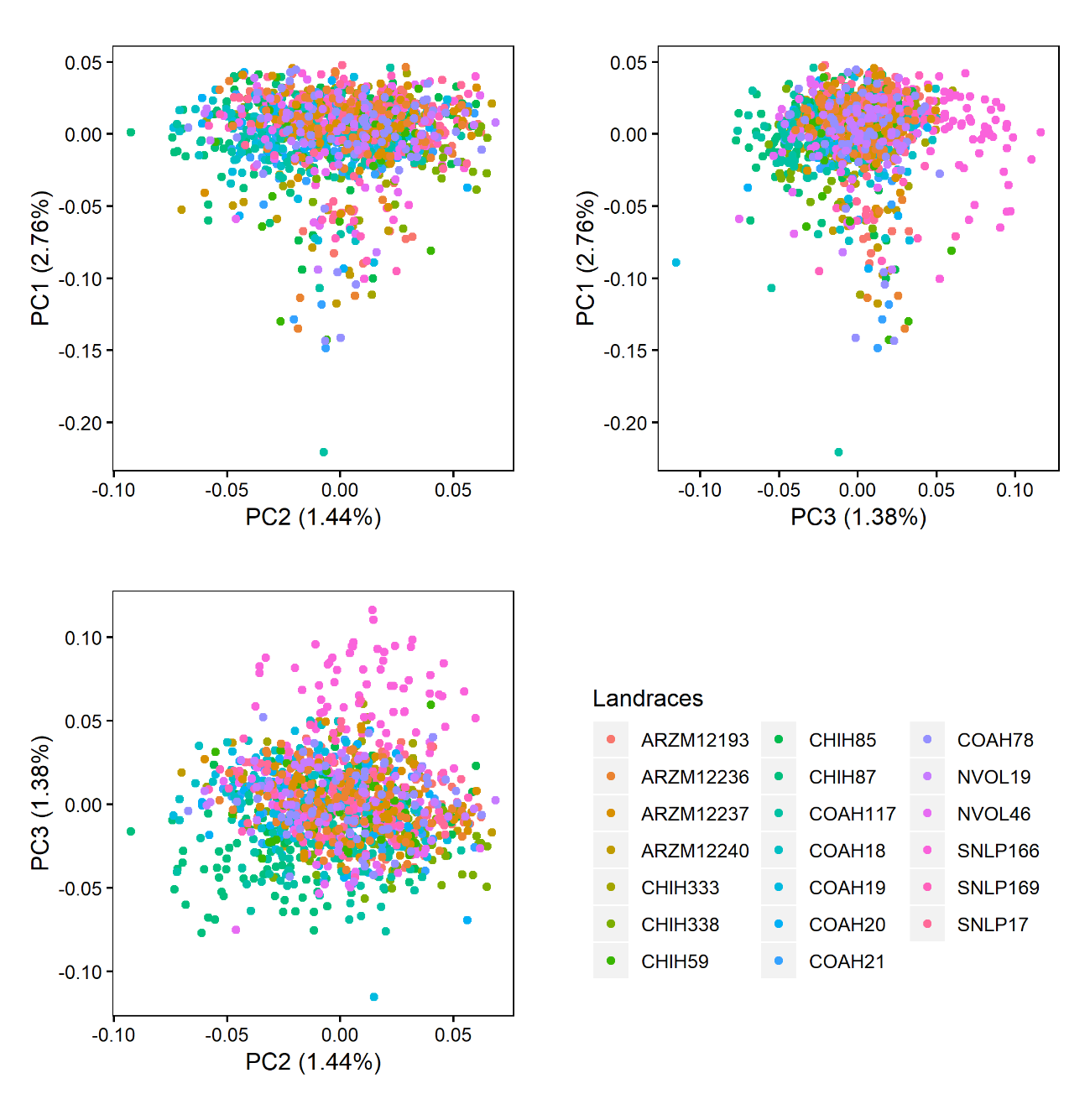


Supplementary Figure 4. Plots of the three first principal components. The 20 landraces families are distinguished by the colors.


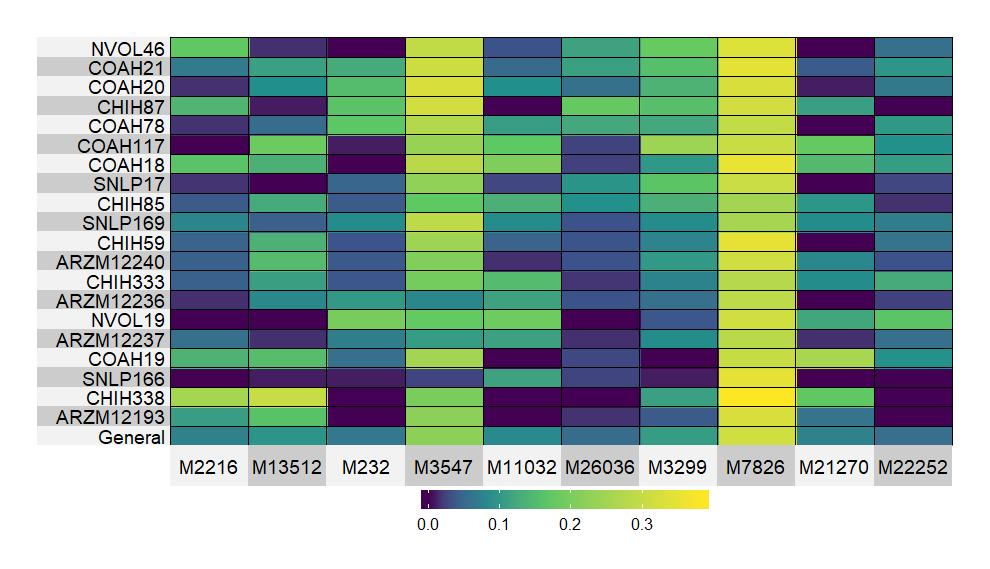
Supplementary Figure 5. Frequency of allele from landrace for the significant markers (columns) by landrace family (rows) and in all the population (general). The frequency of allele from landrace over the entire population are the same values of the MAF presented in Table 4, once the allele from landraces is, as expected, the allele in minor frequency. Landraces are ranked by the higher frequency of superior allele considering the four SNPs where the landrace allele have shown positive effect on the traits (M3547, M11032, M26036, M3299, highlighted by the red rectangle). This rank gives the idea of which landraces are the most promising source for positive variants.


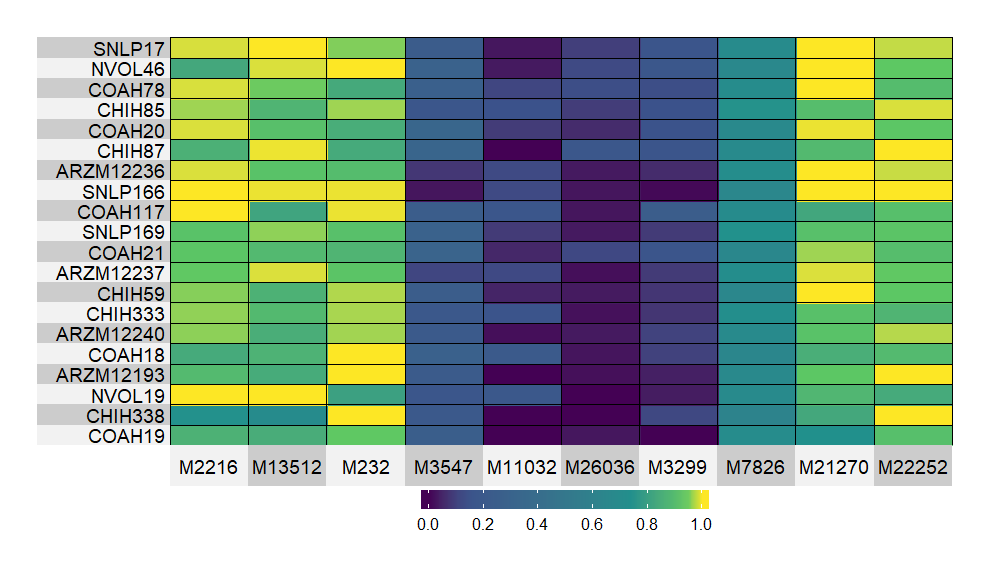
Supplementary Figure 6. Frequency of superior allele (positive effects) for significant markers (columns) by landrace family (rows). Landraces are ranked by the higher frequency of superior allele considering all the 10 SNPs, regardless their origin. This rank gives the idea of which landrace populations are the promising to selection in this breeding population regards the combination with adapted elite genitor.

# Supplementary Table

Table 1: Number of markers, average values for percentage of markers that are missing data, heterozygous, and the minor frequency allele, data presented by chromosome. “Unmapped” groups those markers that were not mapped in any maize nuclear chromosome. The data are shown before (pre-QC) and after (pos-QC) the quality control process detailed at section 2.3 – Material and Methods for Genotypic Data.

| Chr. | N. of markers | | Missing data | | Heterozygous | | MAF (%) | |
| --- | --- | --- | --- | --- | --- | --- | --- | --- |
|  | pre-QC | pos-QC | pre-QC | pos-QC | pre-QC | pos-QC | pre-QC | pos-QC |
| 1 | 4777 | 870 | 8.76 | - | 5.81 | 19.19 | 3.66 | 9.40 |
| 2 | 3841 | 723 | 8.86 | - | 5.56 | 18.61 | 3.56 | 9.08 |
| 3 | 3529 | 662 | 9.39 | - | 5.85 | 19.51 | 3.68 | 9.43 |
| 4 | 3379 | 599 | 8.33 | - | 5.34 | 18.26 | 3.15 | 8.95 |
| 5 | 3646 | 646 | 9.30 | - | 5.53 | 18.78 | 3.58 | 9.19 |
| 6 | 2461 | 423 | 8.75 | - | 5.2 | 18.04 | 3.38 | 8.79 |
| 7 | 2720 | 443 | 8.68 | - | 5.04 | 17.34 | 3.12 | 8.56 |
| 8 | 2951 | 496 | 9.68 | - | 5.24 | 18.90 | 3.41 | 9.16 |
| 9 | 2690 | 427 | 8.85 | - | 5.14 | 19.08 | 3.16 | 9.21 |
| 10 | 2598 | 406 | 10.03 | - | 5.00 | 19.31 | 3.12 | 9.24 |
| Unmapped | 14482 | 0 | 11.60 | - | 5.71 | - | 3.73 | - |
| Total | 47074 | 5695 | 9.06 | - | 5.37 | 18.70 | 3.38 | 9.10 |
